# A hybrid machine learning approach for the personalized prognostication of aggressive skin cancers

**DOI:** 10.1038/s41746-024-01329-9

**Published:** 2025-01-08

**Authors:** Tom W. Andrew, Mogdad Alrawi, Ruth Plummer, Nick Reynolds, Vern Sondak, Isaac Brownell, Penny E. Lovat, Aidan Rose, Sophia Z. Shalhout

**Affiliations:** 1 Translation and Clinical Research Institute, Newcastle University, Newcastle upon Tyne, UK; 2 Department of Plastic and Reconstructive Surgery, Royal Victoria Infirmary, Newcastle Upon Tyne Hospital NHS Foundation Trust (NuTH), Newcastle upon Tyne, UK; 3 Department of Oncology, Newcastle University and Northern Centre for Cancer Care, Newcastle upon Tyne, UK; 4 NIHR Newcastle Biomedical Research Centre & Department of Dermatology, Royal Victoria Infirmary, Newcastle Upon Tyne Hospital NHS Foundation Trust (NuTH), Newcastle upon Tyne, UK; 5 Department of Cutaneous Oncology, Moffitt Cancer Center, and Department of Oncologic Sciences, Morsani College of Medicine, University of South Florida, Tampa, FL USA; 6 Dermatology Branch, National Institute of Arthritis Musculoskeletal and Skin Diseases (NIAMS), National Institutes of Health (NIH), Bethesda, MD USA; 7 Mike Toth Head and Neck Cancer Research Center, Division of Surgical Oncology, Department of Otolaryngology-Head and Neck Surgery, Mass Eye and Ear, Boston, MA USA; 8 Department of Otolaryngology-Head and Neck Surgery, Harvard Medical School, Boston, MA USA

**Keywords:** Translational research, Cancer models, Skin cancer

## Abstract

Accurate prognostication guides optimal clinical management in skin cancer. Merkel cell carcinoma (MCC) is the most aggressive form of skin cancer that often presents in advanced stages and is associated with poor survival rates. There are no personalized prognostic tools in use in MCC. We employed explainability analysis to reveal new insights into mortality risk factors for this highly aggressive cancer. We then combined deep learning feature selection with a modified XGBoost framework, to develop a web-based prognostic tool for MCC termed ‘DeepMerkel’. DeepMerkel can make accurate personalised, time-dependent survival predictions for MCC from readily available clinical information. It demonstrated generalizability through high predictive performance in an international clinical cohort, out-performing current population-based prognostic staging systems. MCC and DeepMerkel provide the exemplar model of personalised machine learning prognostic tools in aggressive skin cancers.

## Introduction

Skin cancers are the most common form of cancer worldwide^1^. Accurate prognostication guides optimal clinical management in skin cancer. Merkel cell carcinoma (MCC) is the most aggressive form of skin cancer with a case fatality rate more than twice that of melanoma^2^. Historically, outcomes have been poor due to its tendency to present in elderly patients at locoregionally advanced stages^3,4^.

The prognostication of MCC is currently based on the features tumor size and spread, as outlined by the American Joint Cancer Committee (AJCC) staging system (Supplementary Table 1)^5,6^. However, this system has notable limitations, as it relies on overall survival endpoints, which, unlike disease specific survival (DSS), fail to account for competing risks and often underestimate survival^7,8^. These constraints in feature selection and survival endpoints reduce the prognostic power of current MCC staging systems, limiting its ability to support complex decision-making in clinical scenarios^9^.

Machine learning (ML) approaches have been shown to enhance the analysis and interpretation of cancer datasets^10–12^. There are distinct advantages to hybrid ML approaches for highly aggressive cancer such as MCC through leveraging limited data, analyzing complex disease biology, and enabling personalized risk stratification to enhance patient outcomes^13^. This approach enables identification of multiple interdependent features within broader real-world dataset, with the potential to uncover complex patterns in disease prognostication.

The limitations of current MCC staging systems, combined with advances in hybrid ML approaches raise the question of whether prognostic accuracy could be improved through personalized approaches. In this context, MCC serves as a valuable model for exploring personalized prognostic tools in aggressive skin cancers. The primary objective of this study was to develop a hybrid ML model tailored to aggressive skin cancers, test it using real-world datasets and create a web-based research tool to help guide precision medicine.

## Results

**Fig. 1:**
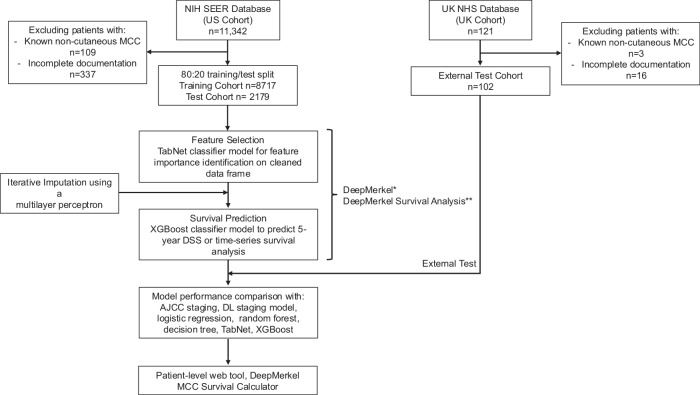
Study design and workflow of patient selection. *DeepMerkel model consists of TabNet/XGBoost framework for 5-year DSS prediction. **DeepMerkel Survival Analysis consists of TabNet/XGBoost framework for time-series DSS prediction. Machine Learning (ML) staging model consists of TabNet/XGBoost framework of staging features only.

The data assembly process is outlined in Fig. 1. 11,342 patients were enrolled into the SEER database with histologically confirmed MCC in the study period. Of these, 109 patients had known non-cutaneous primaries and 377 had missing follow-up data and were therefore excluded. 121 patients with histologically confirmed MCC were identified in the UK data in the study period. Of these, 19 patients were excluded from analysis, 3 patients with known non-cutaneous primary MCC and 16 due to incomplete documentation.

Baseline features of the training cohort, US test and UK test cohorts are presented in Table 1. The median age of US data patients was younger than UK data patients (77 vs 77 vs 80 years, *p* < 0.001) for the training, US test, and UK test cohorts respectively. Most patients were male 64% (6901), white 95.2% (10334), and married 63% (6335). There was no significant difference between T-stage distribution, 82% T1 or T2 in the training, 82% in the US test, and 88% in the UK test cohort (*p* = 0.21). This was also true for nodal involvement at presentation, 31% of the training cohort, compared to 30% in the US test cohort and 34% in the UK test cohort (*p* =·66). 9% of the training, 8% of the US test and 7% of UK test cohort had distant metastasis (*p* = 0.58). Median follow-up time was 29 months in the US test and training cohorts compared to 27 months in the UK test cohort. Kaplan-Meier analysis demonstrated no difference in DSS between the training, US test, and UK test cohorts over 5 years, *p* = 0.34.

**Table 1:** Main characteristics of Merkel cell patients in dataset

Characteristics	Dataset, No. (% of complete data)			
	US SEER Cohort (*n* = 10856)	US Training Cohort (*n* = 8717)	US Test Cohort (*n* = 2179)	UK Test Cohort (*n* = 102)
Age at diagnosis, median (range), y	77 (18–95)	77 (18–95)	77 (18–95)	80 (39–100)
Sex (%)				
Female	3955 (36)	3272 (36)	853 (38)	48 (47)
Male	6901 (64)	5801 (64)	1416 (62)	54 (53)
T Stage				
T0	372 (6)	296 (6)	76 (5)	5 (5)
T1	3849 (57)	3064 (57)	785 (57)	53 (52)
T2	1718 (25)	1360 (25)	358 (26)	37 (36)
T3	419 (6)	343 (6)	76 (5)	5 (5)
T4	403 (6)	312 (6)	91 (7)	4 **(4)**
N Stage				
N0	5962 (69)	4744 (69)	1218 (69)	67 (66)
N1	2478 (29)	1980 (29)	489 (28)	32 (31)
*N2*	172 (2)	132 (2)	40 (2)	3 (3)
*N3*	*36 (* < *1)*	30 ( < 1)	6 (1)	1 (1)
M Stage				
M0	8590 (92)	6840 (91)	1750 (92)	95 (93)
M1	792 (8)	643 (9)	149 (8)	7 (7)
MCC-Specific Mortality (%)				
2- years	2431 (33)	1981 (34)	450 (30)	17 (29)
5- years	2868 (36)	2335 (39)	553 (37)	27 (45)

DeepMerkel a personalized hybrid model consisting of a TabNet/XGBoost framework was developed and compared against the AJCC 8th Edition MCC staging system in predicting DSS. Specifically, a TabNet model was trained for feature selection using 50 epochs, where the training loss decreased steadily over epochs from 17.59 to 0.03. Concurrently, the validation AUC score increased from 0.48 to 1.0, indicating improving model performance on unseen data. Early stopping occurred at epoch 12 with a validation AUC of 0.54, suggesting optimal model performance was achieved at this point. TabNet feature importance analysis revealed that the most influential features for predicting DSS were locally advanced disease spread (1.33%), distant metastasis present (1·24%), and metastatic disease at presentation (1–10%). In addition, non-staging features; patient age, sex, ethnicity and race, marital status, household income, year of diagnosis, level of tumor invasion, tumor site, laterality of lesion, subsequent primary cancer, number of previous primary cancers and timing of regional lymph node involvement were all identified as significant features influencing DSS (Supplementary Note 1). These findings led to the inclusion of a combination of staging and non-staging features in the modified XGBoost classifier (Supplementary Table 2).

Use of a neural network iterative imputation estimator effectively attributed missing values in the collective dataset, demonstrating satisfactory convergence and accurately filling in missing features. The neural network imputation estimated the patient’s age with a Mean Absolute Error (MAE) of 1.5 years and a Root Mean Squared Error (RMSE) of 2.0 years. Similarly, imputed sex and ethnicity exhibited low error rates, with MAE values of 0.05 and 0.1, respectively, indicating accurate estimations. The neural network-based imputation approach achieved consistently low MAE and RMSE values across all features, ensuring the completeness and accuracy of the imputed dataset for subsequent analyzes.

**Fig. 2:**
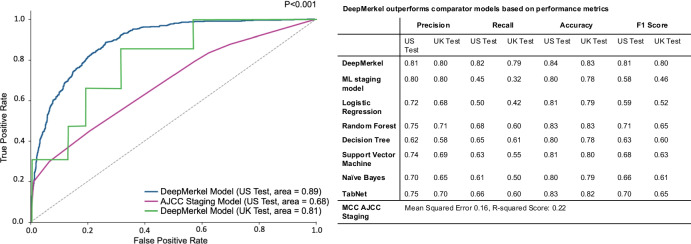
DeepMerkel outperforms other models in predicting DSS. Graphical representation of AUROC comparison of DeepMerkel (US test cohort, blue, AUROC = 0.89), DeepMerkel (UK test cohort, green, AUROC = 0.81), and ML modeling of staging features (purple, AUROC = 0.68) (*p* < 0.001). Tiered AJCC staging has not been represented in the graph (AUROC = 0.55). Table represents performance metrics of comparators models and DeepMerkel.

Results showed DeepMerkel outperformed other machine learning models in both test cohorts: Decision-Tree Classifier, Random Forest Classifier, and Logistic Regression (Fig. 2). DeepMerkel also achieved a significantly higher AUROC score in the US test cohort (0·89) and UK test cohort (0.81) when compared to AJCC Staging (0.55; *p* < 0.001) (Fig. 2). As AJCC staging uses a simple tier-based system to compare staging features more fairly against those included in DeepMerkel we develop the ML Staging Model which includes all staging features including in AJCC without the inclusion of non-staging features but uses the same DeepMerkel framework. DeepMerkel also achieved significantly higher ROC-AUC score when compared to the ML Staging Model, 0.68 (*p* < 0.001). Additionally, DeepMerkel exhibited superior performance across various discrimination thresholds when compared to the current staging system and other machine learning models. (Fig. 2).

**Fig. 3:**
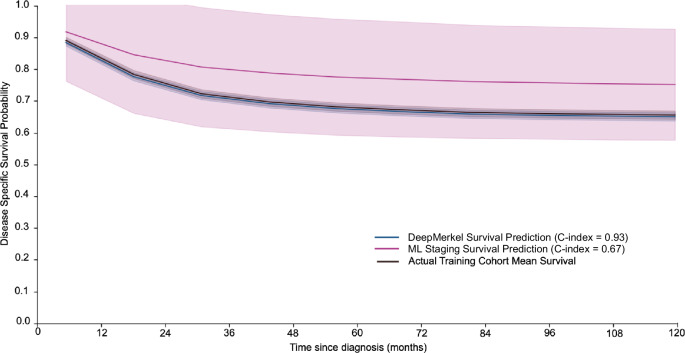
DeepMerkel survival analysis can accurately perform time-series prediction of survival. Kaplan-Meier curve demonstrates the predicted mean survival of DeepMerkel Survival Analysis (blue) compared to the actual mean survival observed (black) (c-index = 0.93). This is compared to predicted mean survival in deep learning modeling of AJCC staging features only (purple) (c-index = 0.67). Mean survival of tiered AJCC staging is not presented in this graph (c-index= 0.51). 95% confidence intervals are demonstrated.

DeepMerkel Survivor Predictor was developed from modifications of the DeepMerkel framework to make time-to-event survival predictions. Use of the developed DeepMerkel Survival Predictor demonstrated superior prognostic performance with a C-index of 0.93 when compared to the DL staging model, 0.67 and tiered AJCC staging, 0.51 (Log-rank Test *p* < 0.001) (Fig. 3). Additionally, DeepMerkel exhibited a low average Brier Score of 0.053, signifying good calibration of predicted probabilities. The D-Calibration score for DeepMerkel did not exceed the threshold of 0·05, suggesting acceptable calibration of survival probabilities. This was also not met by the ML staging model only but was exceeded by AJCC staging.

**Fig. 4:**
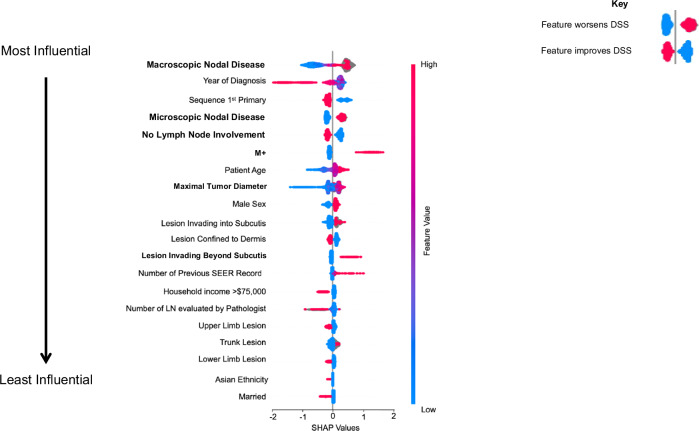
Staging and non-staging features are critical for DeepMerkel performance. Top 20 Shapley values representing feature importance in DSS have been displayed. This has been validated against clinical domain knowledge. Features in bold are staging features, all others are non-staging features.

SHAP values were used to identify the most influential features of the DeepMerkel Survival Predictor against clinical domain knowledge (Fig. 4). Lymph nodal involvement, distant metastasis, patient age, tumor diameter, year of diagnosis and subsequent primary cancer showed the widest range, indicating they had the greatest impact in DSS outcome. Decrease in tumor size and patient age indicated a higher likelihood of survival in the observed instance, whereas patients with multiple previous primary cancers demonstrated the opposite trend. Findings have also shown that the presence of microscopic/macroscopic nodal disease, distant metastasis, male sex, invasion beyond the dermis, and truncal lesions were associated with worse DSS. However, DSS was better if: this was the patient’s first primary cancer, household income exceeded $75,000, the lesion was of the upper/lower limb, the patient was married or of Asian ethnicity (Fig. 4).

**Fig. 5:**
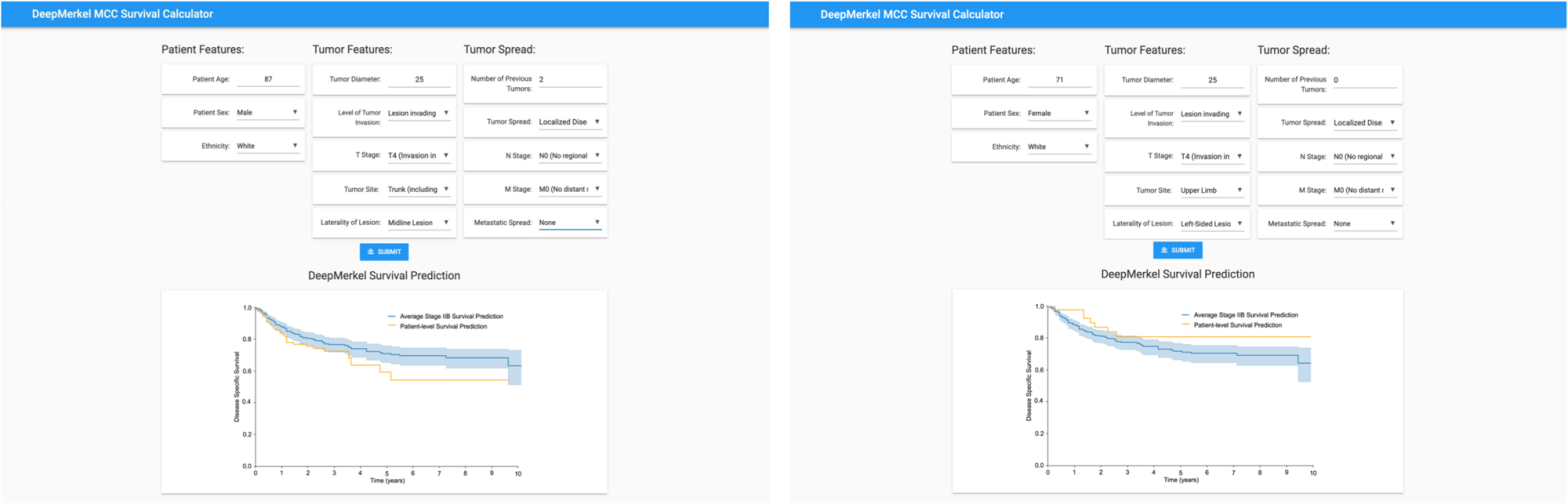
User-friendly DeepMerkel MCC Survival Calculator can predict patient-level DSS. Representative screenshot of two patients with Stage IIb MCC. These Kaplan–meier plots demonstrate a significant difference in DSS based on non-staging features. Blue line = average predicted survival in stage IIb patients (CI included), Orange line- predicted survival of individual based on patient features, tumor.

The DeepMerkel survival calculator was subsequently developed as a real-time clinical web-based tool (Fig. 5). Here, personalized prognostication is derived based on patient features, tumor features and tumor spread. Using this clinical information and the DeepMerkel Survival Predictor a Kaplan–Meier curve plot is generated with predicted survival compared against global predictions based on AJCC stage. Non-staging features substantially influenced survival predictions, as demonstrated in test patients with stage IIB disease (Fig. 5).

## Discussion

Ambiguity in optimal management and poor survival outcomes is exacerbated in highly aggressive skin cancers leading to limitations in clinical developments with traditional investigative approaches^6^. Despite the recent advances in systemic treatment, there aren’t any clinically available MCC survival tools. Disease staging which relies on anatomical observations in tumor size and spread is limited by the survival endpoints used for analysis, features selection and human-defined thresholds for this tiered stratification system. The limitation of current staging emphasizes the unmet clinical need for a more complex ML model which includes non-staging features for a personalized prognostication model.

To enhance the predictive performance and provide comprehensive insights into MCC survival outcomes a hybrid ML model, DeepMerkel, which combines the strengths of TabNet and XGBoost frameworks, was developed. DeepMerkel outperformed current conventional AJCC staging, considered the clinical gold standard^5^. This hybrid model leverages TabNet’s interpretability and XGBoost’s predictive power to handle tabular data and complex interactions effectively. To the best of our knowledge, this is the first study to explore the use of ML in MCC. Results showed DeepMerkel accurately predicted DSS at 5 years; however, differentiating time-to-death provided crucial clinical insight with the potential to impact therapeutic intervention and patient care. To address this need, a modified model of DeepMerkel, DeepMerkel Survival Predictor, was developed using a modified XGBoost approach. This advanced model incorporates time-to-event data, enabling the prediction of not only survival but also the time to death from MCC. DeepMerkel Survival Predictor’s performance metrics were superior to those of current staging systems and alternative ML models, with enhanced calibration, unbiasedness, and stability of survival probabilities.

Previous efforts to use ML to enhance AJCC staging, such as those by Roffman et al., utilized artificial neural networks on non-melanoma skin cancer data, achieving an AUC-ROC of 0.81^14^. Although this did not include MCC, it demonstrated the potential role of ML in identifying high-risk factors in skin cancer. Other groups have reported similar predictive accuracy using XGBoost in cancer registry data^15–17^. However, XGBoost is prone to overfitting^18^, which can compromise performance in specific subgroups. Ma et al. addressed this by integrating Lasso-Cox for feature selection and shrinkage, improving the C-index by 6.16%^19^. DeepMerkel’s hybrid approach was designed to mitigate these risks, showing improved performance compared to other models. Choi et al. combined TabNet and XGBoost in an ensemble model for early determination of meningitis and encephalitis etiologies, achieving similar predictive outcomes to this study with a smaller dataset^20^. Their approach involved duplicating the data sample, potentially limiting external validity. In contrast, DeepMerkel, was tested in two independent hold-out cohorts and demonstrated superior accuracy and generalizability. Other groups have improved model performance by inputting time-series and feature data into survival analysis using a Cox proportional hazard framework combined with XGBoost^19^. DeepMerkel Survival Predictor outperformed these models through iterative training of multiple classifiers enabling the estimation of survival probabilities at discrete time points. Further, DeepMerkel Survival Predictor uses XGBoost as a feature transformer, applied a Kaplan–Meier estimator on the nearest neighbor for survival prediction, and provided non-parametric confidence intervals for survival predictions using bootstrap resampling.

SHAP values were used for model explainability. Multiple non-staging features have been shown to influence MCC survival and disease recurrence^21^. SHAP analysis identified regional lymph node involvement as the most critical independent prognostic predictor. This was true for both macroscopic and microscopic nodal disease, although more pronounced in macroscopic disease due to a higher tumor burden^22^. The number of involved nodes further increased the risk of death, underscoring the importance of sentinel lymph node biopsy in MCC management and informing radiological and surgical interventions^23,24^. Additionally, SHAP analysis showed that distant metastasis significantly influenced DSS, reported to be just 38.2% at 1 year^25^. As systemic therapy evolves, the role of distant metastasis will likely become more crucial in survival predictions. In the current study, non-staging features, in particular increasing age and male gender also correlated with worse DSS, aligning with previous reports of worse DSS in older and male patients^26,27^.

Importantly, SHAP analysis identified a complex relationship between tumor size and level of invasion, highlighting the significance of these factors in survival prediction. Current tumor diameter thresholds, set at 2 cm by human consensus, were optimized through ML approaches, capturing non-linear relationships, and enabling personalized prognostication. Smaller lesions had a greater impact on survival, emphasizing the importance of early diagnosis^28^. Additionally, tumor invasion beyond the dermis into subcutis was shown to be critical, suggesting that invasion depth should be considered a high-risk feature, as is the case for cutaneous squamous cell carcinoma^29^.

Patients included in the present study with subsequent primary MCCs had worse survival outcomes than those presenting with their first primary MCCs, this has been previously documented in all-cause mortality of other primary cancers^30^. Additionally, other studies have also shown that patients with MCC had an increased risk of developing subsequent cancers^31^. Interestingly, chronic lymphocytic leukemia has been shown to substantially increase the risk for polyomavirus DNA-positive MCC^32^. This emphasizes the need for ongoing surveillance among cancer survivors. Additionally, tumors of the trunk were associated with worse DSS, likely due to their potential to invade multiple draining lymph node basins, and the limitation of skin self-assessment. This hypothesis is further supported by the observed correlation between non-married status and worse DSS. Strikingly, patient subsets within high-income households showed better DSS, suggesting that socio-economic factors including: education, healthcare access, and occupational hazards all influence survival. These findings promote the role for intensified clinical skin surveillance based on non-staging and non-clinical risk factors applied to all patients. These social determinants of health (SDOH) were included in the initial model development due to their importance in understanding broader population health trends and disparities. However, SDOH have been removed from the web-based survival tool to ensure clinical decisions remain focused on medical factors that can be actively managed or addressed within the clinical setting.

The objective of this study was to develop a personalized web-based survival research tool to help guide clinical practice. This has been achieved through the integration of the DeepMerkel Survival Calculator. Unlike, current staging systems, which categorize individuals based on global population data, this web application provides individual patient prognoses based on an array of features. This is perhaps best highlighted by looking at individuals of the same stage. We demonstrate that individuals of the same stage may exhibit considerable heterogeneity in survival predictions, resulting in suboptimal clinical management if treated according to stage. This has the potential to lead to under-treatment or over-treatment in the current homogenous conventional staging system. DeepMerkel survival calculator provides personalized survival predictions, encouraging tailored intervention and precision medicine in real-time.

There are limitations associated with this study. Polyomavirus and immune status, which are both important prognostic biomarkers were not analyzed due to their absence in the SEER database and UK sites^33^. Inclusion of these features would likely enhance the model’s accuracy. Year of diagnosis was shown to be a highly influential feature in the model, likely due to advancements in treatment, notably immunotherapy^34^. However, as this was excluded from later model development its effect could not be validated in prospective data collected beyond the study period. Future research should focus on integrating these missing features, co-designing the final website with patients with suitable lived experience of the disease and validating the model in prospective clinical trials before this tool is used in clinical practice.

DeepMerkel can make time-dependent survival predictions providing personalized prognostication and clinical guidance in MCC. This hybrid approach uses applied models which have been specifically adapted and integrated for MCC DSS predictions and has demonstrated generalizability through high predictive performance in an international clinical cohort, out-performing current population-based prognostic staging systems. An individualized web-application survival calculator has been developed to change the current clinical approach to MCC prognostication. Additionally, explainability analysis has revealed new insights into mortality risk factors for this highly aggressive cancer. MCC and DeepMerkel provide the exemplar model of personalized machine learning prognostic tools in aggressive skin cancers.

## Methods

### Data source and study population

Data were collected from the NIH SEER database (US data) and 30 UK NHS hospitals (UK data). The US data included all patients diagnosed with MCC between Jan 1, 2000, and Dec 31, 2020, obtained from the SEER Research Plus Data, 17 registries, using SEER*Stat software (version 8.4.2). The UK data included all patients diagnosed with MCC from Jan 1, 2010, and Dec 31, 2020, from all UK hospitals (Supplementary Note 2). The study excluded participants who had incomplete documentation or presented with known non-cutaneous primary MCC or in situ MCC. During data cleaning and feature selection, we thoroughly reviewed to ensure that only the most accurate and updated features were included in the model. Any redundant or outdated features were excluded to enhance model precision and relevance. All numerical variables were normalized by min–max scaling and used in the process. The primary outcome of the study was DSS, defined as the duration between the diagnosis date and the date of death from MCC. DSS was categorized it into discrete annual intervals from 1 to 10 years to facilitate classification analysis. Patients who were lost to follow-up or remained alive at the time of evaluation were censored. Patients who died within 5 years of initial diagnosis from other causes were censored according to competing risk analysis. Ethics approval was obtained for the UK data through the Newcastle University Dermatology Biobank (REC REF 19/NE/004_Lovat). Access to the SEER database operated under its open access policy. The study was performed in accordance with approved guidelines and regulations for medical research expressed in the Declaration of Helsinki.

### DeepMerkel development

The workflow of data processing and model development is summarized in Fig. 1. The US data was randomly split (60:20:20) into training, validation and holdout test cohorts. The UK data formed a smaller second holdout test cohort to be used in an unbiased manner to assess the final model performance and generalizability. Internal cross-validation ensured that hyperparameter tuning, feature selection, early stopping, and model development were performed independently of the final hold-out 20% SEER test and UK test (approximately 1%) sets. Both test cohorts were not exposed to the model during training, hyperparameter tuning, or validation. These test cohorts were only used to evaluate the final model’s performance after all tuning processes were completed, ensuring an unbiased assessment of model generalizability. Features included in the initial model development are listed in Supplementary Table 2. Data rows were initially excluded if containing any missing values to form a cleaned dataframe. Feature importance analysis was quantified using a custom TabNet classifier model on the cleaned dataframe. Training and validation losses were plotted over epochs, monitoring model convergence. The training loss tracked optimization, while the validation area under the curve (AUC) assessed model discriminative ability.

Correlation matrices were formed based on identified significant features. Features with correlations >0.7 or <−0.7 were excluded to avoid collinearity and feature redundancy. Rows with missing values of <30% and containing DSS were reintroduced to form the imputation-ready dataframe. A Multilayer Perceptron (MLP) serving as the neural network estimator was designed for iterative imputation of missing values. The imputation model was performed solely on the training dataset, with missing values imputed before model training began. The validation dataset was not imputed directly. Instead, any missing values were handled using the imputation model trained on the training data, ensuring the validation data remained independent and unaffected by the imputation process. Similarly, the SEER test set, reserved for final performance evaluation, was not directly imputed. Missing values in the SEER test set were handled using the imputation model trained on the training data, ensuring no information from the test set influenced the training phase.

The random state parameter was set at 0 to ensure reproducibility and consistency across iterations. During the imputation process optimization techniques were employed to fine-tune the neural network model’s performance. Hyperparameter tuning was conducted using grid search with cross-validation. L1 and L2 regularization were applied to prevent overfitting and improve model generalizability. The maximum number of iterations was set to 100, and convergence was defined as achieving a change in the imputed values below 0.001 between successive iterations. The resulting imputed array was then converted back into a data frame structure for further analysis. DeepMerkel was developed as a hybrid model which has been specifically tailored for MCC DSS prediction and consists of deep learning feature selection and a modified XGBoost framework. The SEER training cohort was used for an XGBoost classifier model with the following hyperparameters: a learning rate of 0.1, 100 estimators, a maximum tree depth of 3, and a random state of 42.

Following the development of DeepMerkel a survival analysis was incorporated into the modified framework for time-to-event prediction. The target variable, representing survival duration and DSS, was structured for survival analysis. Time bins for the survival model were defined to capture survival probabilities at 12-month discrete time intervals. To optimize model performance and explore different scale results, we employed an iterative approach, varying the scale parameter for the accelerated failure time loss distribution in the XGBoost survival model. DeepMerkel Survival Analysis, along with logistic regression parameters, were trained using a bootstrap meta-estimator with 20 iterations. The training data were used to monitor model performance, and early stopping rounds were implemented to prevent overfitting. The tuning parameters were chosen using a grid search, based on validation performance determined by the concordance index (c-index), to predict DSS. Model calibration, reflecting predicted versus observed outcomes, was also assessed by visual inspection of calibration plots. Shapley additive explanations (SHAP) were adopted to validate the model against clinical expertize.

Further refinement involved the development of a clinical web application tool, called ‘DeepMerkel Survival Calculator’, enabling the integration of DeepMerkel for real-time prediction and decision support. This research tool enables clinicians to input relevant patient and tumor features and receive instant DSS prediction.

### DeepMerkel comparison

Baseline database categorical features were compared by χ² test, Fisher’s exact test, or two-tailed t-test. DeepMerkel was compared with several commonly employed machine learning models (logistic regression, random forest, decision tree), tiered AJCC staging system, as well as the ‘ML staging model’ which consisted of the same model architecture as DeepMerkel but included staging features only. DeepMerkel model performance was assessed using the metrics: accuracy, precision, recall, F1-Score, ROC-AUC and concordance index comparison. As the tiered AJCC 8th staging is not a classification model this was compared with DeepMerkel with mean squared error and R-squared score. Time-dependent c-indices were calculated for model discrimination and Brier scores for calibration. Survival analyzes were reported with 95% non-parametric confidence intervals. All tests were two-sided with statistical significance set at *p* < 0.05. Statistical analyzes were performed using Python (version 3.9), with relevant packages detailed in Supplementary Note 3. All code and pretrained models will be publicly available on github for the transparency and reproducibility of this study and to provide a benchmark for future studies.

## Supplementary information

Supplementary Information

## Data Availability

Data collected for this study, including individual participant data and a data dictionary defining each field in the dataset, will be made available upon request. The data to be shared will include deidentified participant data and the data dictionary. Related documents, such as the study protocol and statistical analysis plan, will also be available. These data will be accessible beginning from the publication date of this manuscript. Requests for access to the data should be sent to the corresponding author. Access to the data will be granted upon approval of a data access request, which will be reviewed by the authors. Additional restrictions may apply depending on the nature of the analysis and the intended use of the data.

## Code availability

Data processing and analyzes were conducted using Python programming language. The custom code can be shared upon reasonable request to the corresponding author.
