# Supplementary material for: A hybrid machine learning approach for the personalized prognostication of aggressive skin cancers
Source: NPJ Digit Med. 2025 Jan 8;8:15. doi: 10.1038/s41746-024-01329-9 (PMC11711377; doi:10.1038/s41746-024-01329-9)
Supplement: Supplementary file 1 — Supplementary Information [file 41746_2024_1329_MOESM1_ESM.docx]

**Supplementary Information- A Hybrid Machine Learning Approach for the Personalized Prognostication of Aggressive Skin Cancers**

**Supplementary Table 1- MCC Pathological Staging System Table Based on AJCC 8^th^ Edition**

| **Stage** | **Primary Tumor** | **Lymph Node** | **Metastasis** | **TNM** |
| --- | --- | --- | --- | --- |
| **0** | In situ (within epidermis only) | No regional lymph node metastasis | No distant metastasis | Tis  N0  M0 |
| **I** | ≤ 2 cm maximum tumor dimension | Nodes negative by pathologic exam | No distant metastasis | T1  N0  M0 |
| **IIA** | > 2 cm tumor dimension | Nodes negative by pathologic exam | No distant metastasis | T2-T3  N0  M0 |
| **IIB** | Primary tumor invades bone, muscle, fascia, or cartilage | Nodes negative by pathologic exam | No distant metastasis | T4  N0  M0 |
| **IIIA** | Any size / depth tumor | Nodes positive by pathological exam only (microscopic nodal disease) | No distant metastasis | T1-T4  N1a  M0 |
|  | Unknown primary | Nodes positive by clinical exam, and confirmed via pathological exam (macroscopic nodal disease) | No distant metastasis | Tx  N1b  M0 |
| **IIIB** | Any size / depth tumor | Nodes positive by clinical exam, and confirmed via pathological exam (macroscopic nodal disease) | No distant metastasis | T1-T4  N1b  M0 |
|  | Any size / depth tumor | In-transit metastasis* | No distant metastasis | T1-T4  N2  M0 |
| **IV** | Any size / depth tumor | +/- regional nodal involvement | Distant metastasis confirmed via pathological exam | T1-T4  N0-N2  M1 |

* Tumour distinct from the primary lesion and located between the primary lesion and draining regional lymph nodes or distal to the primary lesion.

**Supplementary Note 1- Model Features before and after feature selection**

**Model Features before feature selection**

Age recode with <1 year olds

Sex

Year of diagnosis

PRCDA 2020

Race recode (W, B, AI, API)

Origin recode NHIA (Hispanic, Non-Hisp)

Race and origin recode (NHW, NHB, NHAIAN, NHAPI, Hispanic)

Site recode ICD-O-3/WHO 2008

Behavior code ICD-O-3

TNM 7/CS v0204+ Schema (thru 2017)

TNM 7/CS v0204+ Schema recode

AYA site recode 2020 Revision

Lymphoid neoplasm recode 2021 Revision

ICCC site recode 3rd edition/IARC 2017

SEER Brain and CNS Recode

Site recode ICD-O-3 2023 Revision

Site recode ICD-O-3 2023 Revision Expanded

CS Schema - AJCC 6th Edition

Primary Site - labeled

Primary Site

Histologic Type ICD-O-3

Behavior recode for analysis

Grade Recode (thru 2017)

Grade Clinical (2018+)

Grade Pathological (2018+)

Laterality

Diagnostic Confirmation

ICD-O-3 Hist/behav

ICD-O-3 Hist/behav, malignant

Histology recode - broad groupings

ICCC site recode extended 3rd edition/IARC 2017

Site recode ICD-O-3/WHO 2008 (for SIRs)

Schema ID (2018+)

AJCC ID (2018+)

EOD Schema ID Recode (2010+)

Site recode - rare tumors

Combined Summary Stage (2004+)

Summary stage 2000 (1998-2017)

SEER Combined Summary Stage 2000 (2004-2017)

SEER historic stage A (1973-2015)

Derived EOD 2018 T (2018+)

Derived EOD 2018 N (2018+)

Derived EOD 2018 M (2018+)

Derived EOD 2018 Stage Group (2018+)

Derived AJCC Stage Group, 7th ed (2010-2015)

Derived AJCC T, 7th ed (2010-2015)

Derived AJCC N, 7th ed (2010-2015)

Derived AJCC M, 7th ed (2010-2015)

7th Edition Stage Group Recode (2016-2017)

Derived SEER Cmb Stg Grp (2016-2017)

Derived SEER Combined T (2016-2017)

Derived SEER Combined N (2016-2017)

Derived SEER Combined M (2016-2017)

Derived SEER Combined T Src (2016-2017)

Derived SEER Combined N Src (2016-2017)

Derived SEER Combined M Src (2016-2017)

Derived AJCC Stage Group, 6th ed (2004-2015)

Breast - Adjusted AJCC 6th Stage (1988-2015)

Derived AJCC T, 6th ed (2004-2015)

Derived AJCC N, 6th ed (2004-2015)

Derived AJCC M, 6th ed (2004-2015)

Breast - Adjusted AJCC 6th N (1988-2015)

Breast - Adjusted AJCC 6th M (1988-2015)

Lymphoma - Ann Arbor Stage (1983-2015)

AJCC stage 3rd edition (1988-2003)

SEER modified AJCC stage 3rd (1988-2003)

T value - based on AJCC 3rd (1988-2003)

N value - based on AJCC 3rd (1988-2003)

M value - based on AJCC 3rd (1988-2003)

TNM Edition Number (2016-2017)

RX Summ--Surg Prim Site (1998+)

RX Summ--Scope Reg LN Sur (2003+)

RX Summ--Surg Oth Reg/Dis (2003+)

RX Summ--Surg/Rad Seq

Reason no cancer-directed surgery

Radiation recode

Chemotherapy recode (yes, no/unk)

Scope of reg lymph nd surg (1998-2002)

RX Summ--Reg LN Examined (1998-2002)

Surgery of oth reg/dis sites (1998-2002)

Site specific surgery (1973-1997 varying detail by year and site)

Radiation to Brain or CNS Recode (1988-1997)

RX Summ--Systemic/Sur Seq (2007+)

Months from diagnosis to treatment

Brain Molecular Markers (2018+)

AFP Post-Orchiectomy Lab Value Recode (2010+)

AFP Pretreatment Interpretation Recode (2010+)

B Symptoms Recode (2010+)

Breslow Thickness Recode (2010+)

CA-125 Pretreatment Interpretation Recode (2010+)

CEA Pretreatment Interpretation Recode (2010+)

Chromosome 19q: Loss of Heterozygosity (LOH) Recode (2010+)

Chromosome 1p: Loss of Heterozygosity (LOH) Recode (2010+)

Fibrosis Score Recode (2010+)

Invasion Beyond Capsule Recode (2010+)

Ipsilateral Adrenal Gland Involvement Recode (2010+)

LDH Post-Orchiectomy Range Recode (2010+)

LDH Pretreatment Level Recode (2010+)

LN Head and Neck Levels I-III Recode (2010+)

LN Head and Neck Levels IV-V Recode (2010+)

LN Head and Neck Levels VI-VII Recode (2010+)

LN Head and Neck Other Recode (2010+)

LN Positive Axillary Level I-II Recode (2010+)

Lymph Node Size Recode (2010+)

Major Vein Involvement Recode (2010+)

Measured Basal Diameter Recode (2010+)

Measured Thickness Recode (2010+)

Mitotic Rate Melanoma Recode (2010+)

Number of Cores Positive Recode (2010+)

Number of Cores Examined Recode (2010+)

Number of Examined Para-Aortic Nodes Recode (2010+)

Number of Examined Pelvic Nodes Recode (2010+)

Number of Positive Para-Aortic Nodes Recode (2010+)

Number of Positive Pelvic Nodes Recode (2010+)

Perineural Invasion Recode (2010+)

Peripheral Blood Involvement Recode (2010+)

Peritoneal Cytology Recode (2010+)

Pleural Effusion Recode (2010+)

PSA Lab Value Recode (2010+)

Residual Tumor Volume Post Cytoreduction Recode (2010+)

Response to Neoadjuvant Therapy Recode (2010+)

Sarcomatoid Features Recode (2010+)

Separate Tumor Nodules Ipsilateral Lung Recode (2010+)

Tumor Deposits Recode (2010+)

Ulceration Recode (2010+)

Visceral and Parietal Pleural Invasion Recode (2010+)

EOD Primary Tumor (2018+)

EOD Regional Nodes (2018+)

EOD Mets (2018+)

Prostate Pathological Extension (2018+)

Tumor Size Summary (2016+)

Regional nodes examined (1988+)

Regional nodes positive (1988+)

SEER Combined Mets at DX-bone (2010+)

SEER Combined Mets at DX-brain (2010+)

SEER Combined Mets at DX-liver (2010+)

SEER Combined Mets at DX-lung (2010+)

Mets at DX-Distant LN (2016+)

Mets at DX-Other (2016+)

Breast Subtype (2010+)

ER Status Recode Breast Cancer (1990+)

Derived HER2 Recode (2010+)

Lymph-vascular Invasion (2004+ varying by schema)

CS tumor size (2004-2015)

CS extension (2004-2015)

CS lymph nodes (2004-2015)

CS mets at dx (2004-2015)

CS Tumor Size/Ext Eval (2004-2015)

CS Reg Node Eval (2004-2015)

CS Mets Eval (2004-2015)

CS site-specific factor 1 (2004-2017 varying by schema)

CS site-specific factor 2 (2004-2017 varying by schema)

CS site-specific factor 3 (2004-2017 varying by schema)

CS site-specific factor 4 (2004-2017 varying by schema)

CS site-specific factor 5 (2004-2017 varying by schema)

CS site-specific factor 6 (2004-2017 varying by schema)

Adjusted CS site-specific factor 7 (2004-2017 varying by schema)

CS site-specific factor 8 (2004-2017 varying by schema)

CS site-specific factor 9 (2004-2017 varying by schema)

CS site-specific factor 10 (2004-2017 varying by schema)

CS site-specific factor 11 (2004-2017 varying by schema)

CS site-specific factor 12 (2004-2017 varying by schema)

CS site-specific factor 13 (2004-2017 varying by schema)

CS site-specific factor 15 (2004-2017 varying by schema)

CS site-specific factor 16 (2004-2017 varying by schema)

CS site-specific factor 25 (2004-2017 varying by schema)

CS version input current (2004-2015)

CS version input original (2004-2015)

CS version derived (2004-2015)

EOD 10 - extent (1988-2003)

EOD 10 - nodes (1988-2003)

EOD 10 - size (1988-2003)

Tumor marker 1 (1990-2003)

Tumor marker 2 (1990-2003)

Tumor marker 3 (1998-2003)

Coding system-EOD (1973-2003)

2-Digit NS EOD part 1 (1973-1982)

2-Digit NS EOD part 2 (1973-1982)

2-Digit SS EOD part 1 (1973-1982)

2-Digit SS EOD part 2 (1973-1982)

Expanded EOD(1) - CP53 (1973-1982)

Expanded EOD(2) - CP54 (1973-1982)

Expanded EOD(1,2) - CP53,54 (1973-1982)

Expanded EOD(3) - CP55 (1973-1982)

Expanded EOD(4) - CP56 (1973-1982)

Expanded EOD(5) - CP57 (1973-1982)

Expanded EOD(6) - CP58 (1973-1982)

Expanded EOD(7) - CP59 (1973-1982)

Expanded EOD(8) - CP60 (1973-1982)

Expanded EOD(9) - CP61 (1973-1982)

Expanded EOD(10) - CP62 (1973-1982)

Expanded EOD(11) - CP63 (1973-1982)

Expanded EOD(12) - CP64 (1973-1982)

Expanded EOD(13) - CP65 (1973-1982)

EOD 4 - extent (1983-1987)

EOD 4 - nodes (1983-1987)

EOD 4 - size (1983-1987)

COD to site recode

COD to site rec KM

COD to site recode ICD-O-3 2023 Revision

COD to site recode ICD-O-3 2023 Revision Expanded (1999+)

Vital status recode (study cutoff used)

Sequence number

First malignant primary indicator

Primary by international rules

Record number recode

Total number of in situ/malignant tumors for patient

Total number of benign/borderline tumors for patient

Race recode (White, Black, Other)

Age recode with <1 year olds and 90+

Age recode with single ages and 85+

Age recode with single ages and 90+

Race/ethnicity

IHS Link

SS seq # - mal+ins (most detail)

SS seq # 1975+ - mal+ins (most detail)

SS seq # 1992+ - mal+ins (most detail)

SS seq # 2000+ - mal+ins (most detail)

Site - mal+ins (most detail)

SS seq # - mal (most detail)

SS seq # 1975+ - mal (most detail)

SS seq # 1992+ - mal (most detail)

SS seq # 2000+ - mal

Site - malignant

Patient ID

Type of Reporting Source

Marital status at diagnosis

CoC Accredited Flag (2018+)

Median household income inflation adj to 2021

Rural-Urban Continuum Code

Race and origin (recommended by SEER)

Race and origin recode (NHW, NHB, NHAIAN, NHAPI, Hispanic)~PRCDA 2020

**Supplementary Table 2: Model Features after feature selection**

| **Feature Group** | **Category** | **Feature Name** |
| --- | --- | --- |
| Patient Feature | Patient Age | Patient Age |
|  | Patient Sex | Male |
|  |  | Female |
|  | Ethnicity and Race | Unknown |
|  |  | White |
|  |  | Black |
|  |  | South Asian |
|  |  | East Asian |
|  |  | South-East Asian |
|  |  | Other Asian |
|  |  | Native American |
|  |  | Oceania/Pacific Islander |
|  | Marital Status | Unknown |
|  |  | Single |
|  |  | Married |
|  |  | Divorced/Separated |
|  |  | Widowed |
|  | Household Income | Unknown |
|  |  | <$35,000 |
|  |  | $35,000 - $39,999 |
|  |  | $40,000 - $44,999 |
|  |  | $45,000 - $49,999 |
|  |  | $50,000 - $54,999 |
|  |  | $55,000 - $59,999 |
|  |  | $60,000 - $64,999 |
|  |  | $65,000 - $69,999 |
|  |  | $70,000 - $74,999 |
|  |  | >$75,000 |
| Tumor Feature | Maximal Tumor Diameter | Maximal Tumor Diameter |
|  | Year of diagnosis | Year of diagnosis |
|  | Level of Tumor Invasion | Lesion confined to dermis |
|  |  | Lesion invading into SC (but not beyond) |
|  |  | Lesion invading into adjacent structures |
|  |  | Lesion invading into cartilage or bone |
|  |  | Lesion invading with skin deposit |
|  | T-stage | Tx |
|  |  | Tis |
|  |  | T1 |
|  |  | T2 |
|  |  | T3 |
|  |  | T4 |
|  | Tumor Site | External ear |
|  |  | Lip (cutaneous) |
|  |  | Scalp and Neck |
|  |  | Unspecified facial lesion |
|  |  | Upper Limb |
|  |  | Lower Limb |
|  |  | Trunk |
|  |  | Unspecified skin lesion |
|  |  | Unknown primary |
|  | Laterality of lesion | Unknown |
|  |  | Laterality-left sided primary |
|  |  | Right sided lesion |
|  |  | Midline lesion |
|  |  | Bilateral lesion |
| Tumor Spread | MCC AJCC Stage | Stage 0 |
|  |  | Stage I |
|  |  | Stage IIA |
|  |  | Stage IIB |
|  |  | Stage IIIA |
|  |  | Stage IIIB |
|  |  | Stage IV |
|  | Subsequent primary cancer | First primary cancer |
|  |  | Subsequent primary cancer |
|  | Number of previous primary cancers | Number of previous primary cancers |
|  | Tumor spread | Localized disease |
|  |  | Regional disease |
|  |  | Distant disease |
|  | N-stage | Nx |
|  |  | N0 |
|  |  | N1a (microscopic nodal disease) |
|  |  | N1b (macroscopic nodal disease) |
|  |  | N2 (in-situ metastasis) |
|  | Timing of regional lymph node involvement | At initial diagnosis |
|  |  | Any point in follow-up after initial diagnosis |
|  | M-stage | M0 |
|  |  | M1 |
|  | Metastatic spread | None |
|  |  | Bone Metastasis |
|  |  | Brain Metastasis |
|  |  | Liver Metastasis |
|  |  | Lung Metastasis |
|  | Axillary node involvement | None |
|  |  | Level I lymph node involved |
|  |  | Level II lymph node involved |
|  |  | Level III lymph node involved |
|  |  | Level I&II lymph node involved |
|  |  | Level I&III lymph node involved |
|  |  | Level II&III lymph node involved |
|  |  | Level I,II&III lymph node involved |

**Supplementary Note 2- List of Hospitals included in the UK Cohort**

Alnwick Infirmary

Alston Hospital

Bensham Hospital

Berwick Infirmary

Bishop Auckland Hospital

Blyth Hospital

Chester-le-Street Hospital

Cockermouth Hospital

Cumberland Infirmary

Darlington Memorial Hospital

Freeman Hospital

Hexham General Hospital

Haltwhistle Hospital

Keswick Hospital

North Tyneside General Hospital

Penrith Hospital

Queen Elizabeth Hospital

Richardson Hospital

Rothbury Hospital

Royal Victoria Infirmary

Sedgefield Hospital

Shotley Bridge Hospital

South Tyneside Hospital

Sunderland Royal Hospital

University Hospital of North Durham

Wansbeck General Hospital

Weardale Hospital

West Cumberland Hospital

Wigton Hospital

Workington Hospital

**Supplementary Note 3- List of Python Packages**

Pandas: 2.2.2

Numpy: 1.26.4

Shap: 0.45.1

Tensorflow: 2.16.1

Keras: 3.3.3

Lifelines: 0.28.0

XGBoost: 2.0.3

Scikit-learn (sklearn): 1.5.0

Matplotlib: 3.9.0

XGBSE: 0.2.3

PyTorch: 2.3.0

PyTorch TabNet: 4.1.0

Scikit-Survival: 0.22.2
